# Spatio-Temporal Analysis of Suicide-Related Emergency Calls

**DOI:** 10.3390/ijerph14070735

**Published:** 2017-07-06

**Authors:** Miriam Marco, Antonio López-Quílez, David Conesa, Enrique Gracia, Marisol Lila

**Affiliations:** 1 Department of Social Psychology, University of Valencia, Avda. Blasco Ibáñez, 21, 46010 Valencia, Spain; enrique.gracia@uv.es (E.G.); marisol.lila@uv.es (M.L.); 2 Department of Statistics and Operations Research, University of Valencia, Dr. Moliner, 50, 46100 Burjassot, Spain; antonio.lopez@uv.es (A.L.-Q.); david.v.conesa@uv.es (D.C.)

**Keywords:** bayesian modeling, disease mapping, police calls-for-service, seasonality, social epidemiology

## Abstract

Considerable effort has been devoted to incorporate temporal trends in disease mapping. In this line, this work describes the importance of including the effect of the seasonality in a particular setting related with suicides. In particular, the number of suicide-related emergency calls is modeled by means of an autoregressive approach to spatio-temporal disease mapping that allows for incorporating the possible interaction between both temporal and spatial effects. Results show the importance of including seasonality effect, as there are differences between the number of suicide-related emergency calls between the four seasons of each year.

## 1. Introduction

Suicide is a global health problem that is receiving increasing interest worldwide [1]. According to the World Health Organization (WHO), in 2012, there were over 800,000 suicide deaths in the world, and suicide was the second leading cause of death after road traffic injuries in young adults [2]. In Spain, where this study was conducted, 3602 people died by suicide in 2015, which corresponds to the 24% of deaths by external causes [3]. The numbers would even increase if we take into account the suicide attempts where the victim finally survives. These data highlight the necessity of further analyzing the different mechanisms that may be involved in this problem.

Although previous research has mainly focused on individual and familiar characteristics of suicide, an increasing number of studies have suggested that spatial patterns would also have an impact in suicide risk, in other words, that suicide would not be randomly distributed across the space, but subjected to underlying spatial patterns [4,5,6,7,8,9,10,11,12,13]. In addition, other studies have shown the seasonality of suicide: previous research suggests that the prevalence of suicide deaths are not constant, but varies during the year [14,15,16,17,18]. Specifically, these studies have shown that there is a peak of suicide cases in spring and summer, and the findings are similar independently of the study country. Indeed, linking both ideas of area patterns and seasonality would result in the most appropriate approach to conduct an ecologic study of suicides [4,10].

The Bayesian approach has turned into an appropriate choice when dealing with spatio-temporal disease mapping models in the public health context [19,20,21,22], although its use for the analysis of suicide-deaths is more recent but already productive [4,5,8,12,13,23,24,25,26,27,28]. A good comparison of the most relevant spatio-temporal disease mapping approaches can be found in this special issue [29].

The focus of this work is to model the spatio-temporal distribution of suicide-related emergency calls (including suicide deaths and suicide attempts) in the city of Valencia (Spain), in particular by means of an autoregressive approach to spatio-temporal disease mapping that brings together ideas from autoregressive time series in order to link information in time and from the spatial modeling context to link information in space [30]. Although other studies have also used calls to analyze different crime and police intervention outcomes [31,32,33], the main focus here is in showing that the inclusion of the effect of seasonality can help to understand the temporal pattern of suicide risks.

## 2. Materials and Methods

### 2.1. Emergency Police Calls in Valencia City

This study was conducted in Valencia (Spain), which is the third largest city in the country with a population of 736,580 inhabitants. The census block group, the smallest administrative unit available, was used as a proxy of neighborhood. In particular, the city of Valencia is divided into 552 census block groups. The population of the census block groups ranges from 630 to 2845, with an average of 1334 residents.

The outcome variable of interest was the number of suicide-related emergency calls. Valencia Police Department and its call service (number 092 in Spain) provided information about all the calls they received requiring police intervention in the city of Valencia. From the entire database, we selected the calls informing of a death by suicide (142 calls) and those informing of a suicide attempt (6395 calls), 6537 being the total number of suicide-related calls. The address where the call was made was geocoded in order to keep track of the census block group where it was produced.

All the suicide-related calls analyzed had been obtained during a seven year period (from 2010 to 2016), providing the possibility of capturing any possible temporal trends. Each year was also divided into 3-month periods, where period 1 starts in January and lasts until the end of March, period 2 comprises from April to June, period 3 from July to September, and period 4 from October to December. The outcome variable was described in those 28 periods allowing the exploration of the possible effect of seasonality.

Table 1 presents summary statistics for the counts of suicide-related emergency calls aggregated both at global and annual scales.

**Figure 1:**
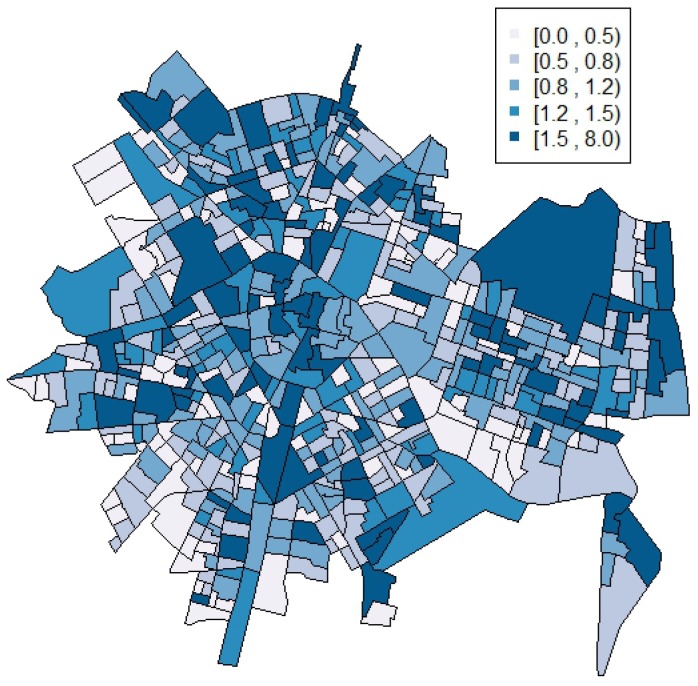
Map of the standardized suicide-related emergency calls ratios in Valencia census block groups during the whole period analyzed (2010–2016).

As it can be appreciated, an aggregated total of 6537 calls was reported and geocoded, being the range of the number of calls for each census block group from 0 up to 70. The standardized suicide-related emergency calls ratio (calculated as the received calls divided by the expected calls by census block group) for all census block group ranges from a minimum of 0.00 to a maximum of 7.38. Note that, as usual in the context of small area disease mapping, there are census block groups for which standardized incidence ratios are zero, the reason underneath being that there are census block groups with zero number of calls. The percentage of census block groups with zero frequency was 0.5% for the whole study period. This percentage increased to 33% when taking into account yearly periods and up to 70.4% when taking into account the 28 quarterly periods. The map of the standardized suicide-related emergency calls ratio can be seen in Figure 1. It is worth noting that the cut-offs in all the figures were selected to provide symmetric intervals and a balanced number of units per interval rounded to one decimal.

### 2.2. Spatial Disease Mapping

A first approach to describe this dataset was to conduct a purely spatial model in line with [5,23,25,34,35]. The most popular one was due to [36], and it is based on considering the observed number of calls as conditionally independent Poisson variables and then linking them via a Poisson regression with two random effects. In particular, if $O_{i}$ represents the observed number of suicide-related emergency calls at the census block group *i*, then:

(1) $$O_{i}∼Poisson\left(E_{i}\exp\left(\eta_{i}\right)\right),\;i=1,\ldots ,552,$$

where $E_{i}$ is a quantity that accounts for the expected number of calls in census block group *i*, that is

$$E_{i}=\mathrm{Population}\;\mathrm{living}\;\mathrm{at}\;\mathrm{census}\;\mathrm{block}\;\mathrm{group}\;i\times \frac{\mathrm{Total}\;\mathrm{number}\;\mathrm{of}\;\mathrm{calls}}{\mathrm{Total}\;\mathrm{population}\;\mathrm{in}\;\mathrm{Valencia}}\;\;\;,$$

and $\eta_{i}$ is the log relative risk that takes into account the spatial effects as

(2) $$\eta_{i}=\mu +\phi_{i}+\theta_{i}$$

μ being the intercept, ϕ a spatially structured random effect, and θ the unstructured random effect.

As stated in [36], the unstructured spatial effect θ is modeled by means of independent identically distributed Gaussian random variables $N(0,\sigma_{\theta }^{2})$, while the structured spatial effect ϕ is considered to be a conditional spatial autoregressive model [36] in order to reflect the spatial neighborhood relationships:

(3) $$\phi_{i}|\phi_{-i}∼N\left(\frac{1}{n_{i}}\sum_{j∼i}\phi_{j},\frac{\sigma_{\phi }^{2}}{n_{i}}\right),$$

where $n_{i}$ is the number of neighboring areas of census block group *i*, $\phi_{-i}$ indicates the values of the ϕ vector except for the *i*th component, the expression j∼i denotes all units *j* that are neighbors of census block group *i*, and $\sigma_{\phi }$ is the standard deviation parameter.

To finally set up the model, and taking into account that in this work the inferential approach was made under the Bayesian paradigm, any information about the unknown parameters was expressed in probabilistic terms via the so-called prior distributions. In particular, an improper uniform distribution was assigned for μ, while for the hyperparameters of $\sigma_{\theta }$ and $\sigma_{\phi }$, prior distributions of standard deviations were uniform distributions $\sigma_{\phi },\sigma_{\theta }∼U\left(0,1\right)$.

### 2.3. Spatio-Temporal Disease Mapping: Annual Data

Incorporating the temporal effect in the context of disease mapping has been a matter of interest for researchers lately. Anderson and colleagues [29] have compared many of them looking for good behaviors in terms of ability to fit and computational effort. In this work, based on [29], comments about its good behavior in terms of fitting, the model proposed by [30] was used. Indeed, previous studies have shown that this approach can provide a very good fit in many complex situations, in particular those involving relevant epidemiological outcomes [29,37,38].

In particular, the number of suicide-related police calls-for-service in each census block group in the seven years of the study, $O_{it}$, were modeled as conditionally independent Poisson distributions

(4) $$O_{it}∼Poisson\left(E_{it}\exp\left(\eta_{it}\right)\right),\;i=1,\ldots ,552,\;t=1,\ldots ,7,$$

where $E_{it}$ is the expected number of calls in census block group *i* during year *t* and $\eta_{it}$ is the log relative risk.

The spatio-temporal effect is included in the model via the $\eta_{it}$. The proposed approach of [30] cleverly combines autoregressive time series and spatial modeling by means of a spatio-temporal structure in which the relative risks are both spatially and temporally dependent. For the first annual period, the relationship is:

(5) $$\eta_{i1}=\mu +\alpha_{1}+{\left(1-\rho^{2}\right)}^{-1/2}\cdot \left(\phi_{i1}+\theta_{i1}\right)\;,$$

while for the remaining periods is

(6) $$\eta_{it}=\mu +\alpha_{t}+\rho \cdot \left(\eta_{i(t-1)}-\mu -\alpha_{t-1}\right)+\phi_{it}+\theta_{it}\;.$$

It is worth noting that, in both equations, $\alpha_{t}$ represents the mean deviation of the risk in year *t*, ρ represents the temporal correlation between years (that is, the temporal correlation between the spatial effects of each year), and $\phi_{it}$ and $\theta_{it}$ refer to the structured and unstructured spatial random effects of each year, respectively. With respect to the structure for $\alpha_{t}$, the choice was a conditional autoregressive temporal model depending on the parameter $\sigma_{\alpha }$, while the unstructured spatial effect is also modeled by means of independent identically distributed Gaussian random variables and the structured spatial effect is considered to be a conditional spatial autoregressive model.

As previously commented, the final set up of the model consists of assigning the priors. In this case, the selection was an improper uniform distribution for μ, uniform over the whole space for the autoregressive term ρ∼U(−1,1), and uniform distributions for the three standard deviations involved $\sigma_{\alpha },\sigma_{\phi },\sigma_{\theta }∼U\left(0,1\right)$.

### 2.4. Spatio-Temporal Disease Mapping: Quarterly Data

As the number of observed suicides could also be seasonal, a spatio-temporal model similar to the previous one but using trimesters as time units was also considered. During the analyzed period, there were 28 trimesters, and for each one, the number of suicide-related police calls-for-service in each census block group was also expressed as

(7) $$O_{it}∼Poisson\left(E_{it}exp\left(\eta_{it}\right)\right),\;i=1,\ldots ,552,\;t=1,\ldots ,28\;.$$

In the same manner as before, the spatio-temporal effect is included in the model via the $\eta_{it}$ by means of [30], but including an additional quarterly effect. Indeed, for the first trimester of 2010, the relationship is now expressed as:

(8) $$\eta_{i1}=\mu +\alpha_{1}+\beta_{1}+{\left(1-\rho^{2}\right)}^{-1/2}\cdot \left(\phi_{i1}+\theta_{i1}\right),$$

while for the remaining periods is

(9) $$\eta_{it}=\mu +\alpha_{t}+\beta_{q(t)}+\rho \cdot \left(\eta_{i(t-1)}-\mu -\alpha_{t-1}\right)+\phi_{it}+\theta_{it}.$$

Note that, similarly to the previous model, in both equations, $\alpha_{t}$ represents the mean deviation of the risk in trimester *t*, ρ represents the temporal correlation between periods, and $\phi_{it}$ and $\theta_{it}$ refer to structured and unstructured spatial random effects of each trimester, respectively. In addition, and in order to express the effect of the four seasons of each year, $\beta_{q(t)}$ represents now the mean deviation of the risk at season q(t). The fourth trimester was selected as the reference one, and the remaining three were compared to it.

Again, assigning the priors is the last step to complete the models. The selection here was an improper uniform distribution for μ, uniform over the whole space for the autoregressive term ρ∼U(−1,1), uniform distributions for the three standard deviations involved $\sigma_{\alpha },\;\sigma_{\phi },\;\sigma_{\theta }∼U\left(0,1\right)$ and Gaussian distributions with large variance for the parameters of the fixed effect $\beta_{1},\;\beta_{2},\;\beta_{3}∼N$(0, 10,000).

A simplified version of this model in which the correlation between periods, ρ, is zero (and so, there is no autoregressive term explaining interactions between space and time) was also considered. This model was analyzed to validate that the extra complexity added by including the interaction between space and time is really worthwhile.

### 2.5. Statistical Inference

As usual in this context, the resulting hierarchical Bayesian model containing all the information about the suicides has no closed expression for the posterior distribution of all the parameters, and so numerical approximations are needed. Computation of posterior probability distributions is not always easy to deal with. For many years, the computational challenge of obtaining posterior distributions has been one of the main issues for not using Bayesian statistics. However, nowadays, this task has been simplified by the increasing capacity of computers together with the development of simulation methodologies based on Monte Carlo sampling and Markov Chain Monte Carlo (MCMC) methods (see, for instance, [39] for a good review on the subject).

MCMC methods can be implemented in many statistical packages. In this paper, MCMC was performed through WinBUGS [40], a statistical software that provides a simple implementation of a great number of complex statistical models. The WinBUGS code of the final model can be found in the Supplementary Materials Section S1. In particular, three chains with 50,000 iterations for each chain were generated, and the first 10,000 for each chain were discarded as burn-in. Convergence of all the chains was assessed by visual inspection of simulated chains and by means of the Brooks–Gelman–Rubin statistic and the effective sample size [41].

Most of the above presented methods are not comparable in terms of useful model selection criteria. For those cases in which there were different comparable models, the Deviance Information Criterion (DIC) [42] was used to compare among them. As other similar criteria, it weighs up the goodness-of-fit and the complexity of the selected model, but, more importantly, it has good behavior when comparing models whose posterior distribution has been approximated by MCMC. The smaller the DIC, the better the fit.

## 3. Results

**Figure 2:**
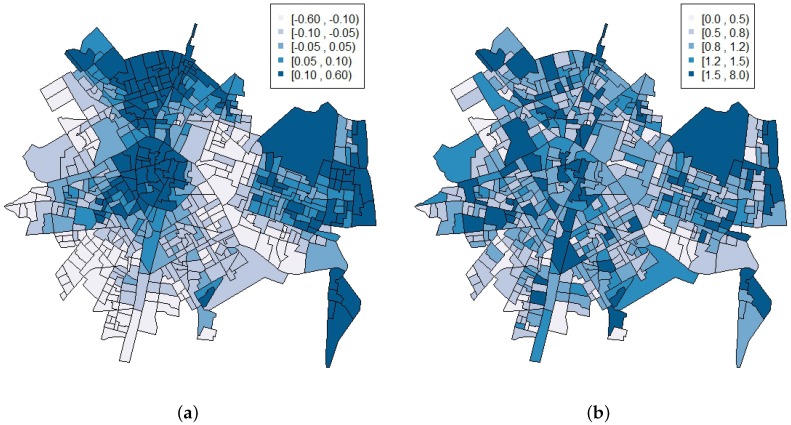
(**a**) spatial effect; and (**b**) relative risks of the pure spatial model.

Table 2 shows the summary statistics along with the credible intervals of the posterior distributions of the parameters of the pure spatial model in Section 2.2, while Figure 2 shows its corresponding spatial effect ϕ and the relative risk for each census block group. Note that the spatial effect is not as important as the heterogeneity effect ($\sigma_{\phi }=0.335$ and $\sigma_{\theta }=0.478$, respectively). However, more importantly, although this spatial effect is relevant and the relative risk for each census block group shows a smoother version of the standardized incidence ratios presented in Figure 1, it is worth mentioning that this pure spatial model does not incorporate any information about any possible time trend.

**Figure 3:**
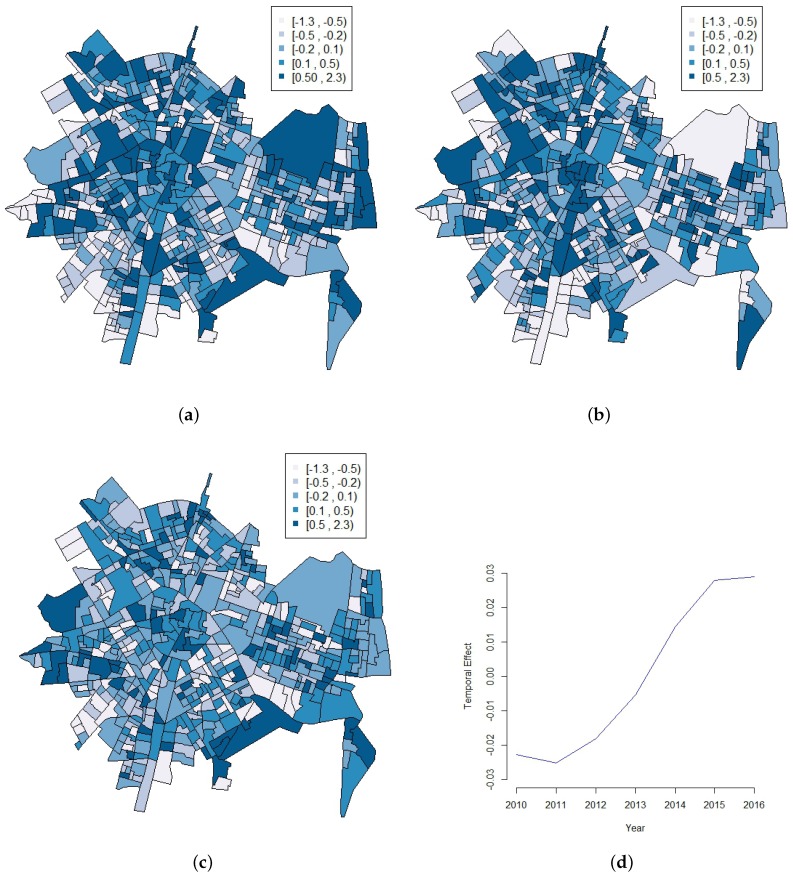
(**a**–**c**): spatial effect for the years 2010, 2013 and 2016, respectively; (**d**): temporal effect during the period (2010–2016).

In order to reflect the possible year effect on the number of calls for each census block group, Table 3 resumes the posterior distributions of the parameters of the spatio-temporal model with annual data introduced in Section 2.3. In the same way as the pure spatial model, the heterogeneity effect ($\sigma_{\theta }=0.512$) has a greater weight than the spatial effect ($\sigma_{\phi }=0.272$). In addition, there are two important things to be noted here, namely, the strong relevance of the autoregressive term (ρ is nearly 0.7), and also the importance of the year term. Nevertheless, this temporal effect (α) can be more clearly appreciated when observing Figure 3d, where an increasing trend of the number of calls for each census block group is clearly marked. This can also be observed in Table 1, where the number of calls increase over the years. Figure 3 also shows the spatial effect (ϕ) for three particular years 2010, 2013, and 2016, showing different patterns for the three years.

Anyhow, taking into account that the temporal effect has resulted in being relevant, a model incorporating the possible seasonality inside years was also analyzed. In particular, Table 4 resumes the posterior distributions of the parameters of the spatio-temporal model with quarterly data introduced in Section 2.4. The convergence of the parameters of the model was properly good, as showed in the Supplementary Materials Section S2. Posterior distributions of the parameters describing the fixed quarterly effect show that the number of calls is larger in the second and third trimester.

**Figure 4:**
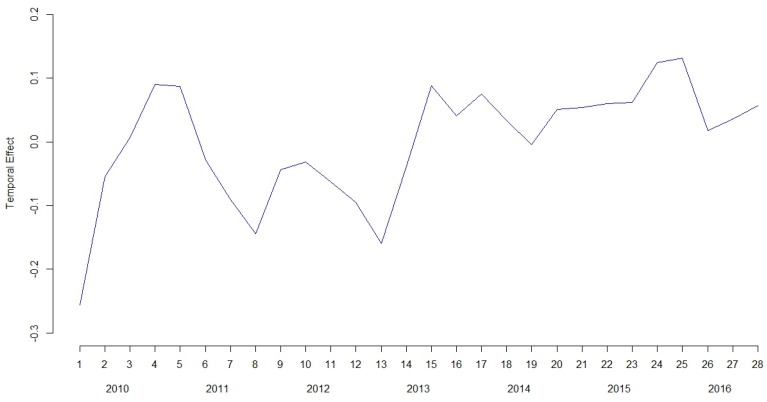
Temporal effect for each trimester during the whole 2010–2016 period analyzed.

Again, results in Table 4 show a higher effect of the heterogeneity parameter ($\sigma_{\theta }=0.359$) compared to the spatial structured term ($\sigma_{\phi }=0.160$), as well as a strong relevance of the autoregressive term (ρ is around 0.9). However, more importantly, the temporal term (without the quarterly effect) now becomes more relevant, shown by a higher value of the autoregressive standard deviation $\sigma_{\alpha }$. The resulting temporal effect (α) presented at Figure 4 clearly shows a different behavior (increasing trend but with more ups and downs) than the one observed at Figure 3d.

**Figure 5:**
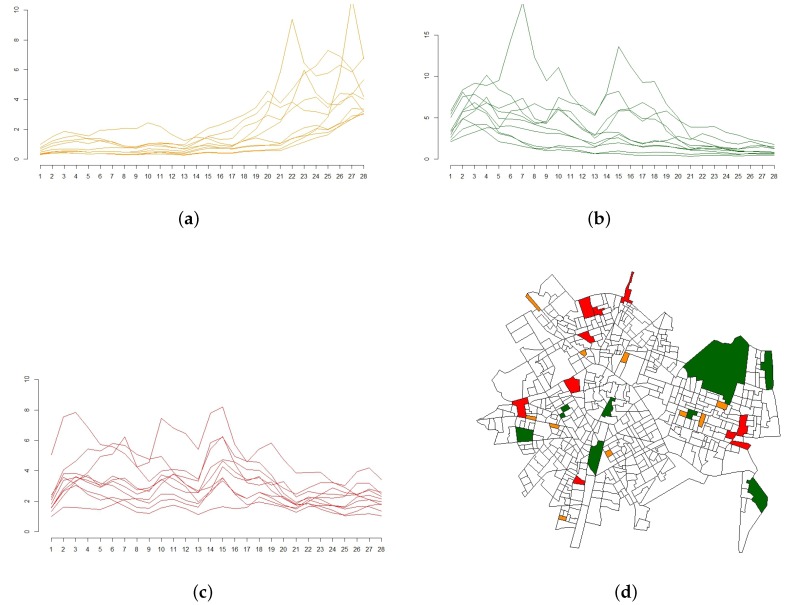
Changes in relative risks for three kind of census block groups in the city of Valencia. (**a**): Relative risks of census block groups with an increasing trend; (**b**): Relative risks of census block groups with a decreasing trend; (**c**): Relative risks of census block groups with permanent high risk; (**d**): Changes in relative risk: location of the selected census block groups in the city of Valencia (in orange those with an increasing trend, in green those with a decrease in the tendency, and in red those where the relative risk was always high).

An added bonus when analyzing this kind of spatio-temporal model is the possibility of describing the temporal trend of each census block group along the whole period. Figure 5 shows the relative risks for three kinds of census block groups (in orange those with an increasing trend, in green those with a decrease in the tendency, and in red those where the relative risk was always high) and their relative position in the city of Valencia.

Finally, and in order to validate that the extra complexity added by including the interaction between space and time is really worthwhile, this autoregressive spatio-temporal model with quarterly data was compared with a simplified version of this model in which the correlation between periods, ρ, is zero. DICs [42] obtained in each case (25,752 in the latter and 24,577 in the former) show that the autoregressive part must be considered.

## 4. Discussion

This study has explored the importance of including possible trends in the context of suicide-related emergency calls. After describing in Section 2 an autoregressive approach to spatio-temporal disease mapping that links information in time and in space, this work has presented how different non-comparable models can show different features from the same dataset when time is considered.

Results have showed that suicide-related emergency calls are spatially patterned. This is in line with previous research that also suggested the unequal distribution of suicide across areas [4,5,6,7,8,9,10,11,12,13]. However, more interestingly, results also indicate that suicide-related emergency calls have a quarterly effect, with a peak of calls in the second (April to June) and in the third trimester (July to September), and a decrease in the other trimesters. These results are in line with previous research that has found higher suicide rates in spring and summer [14,15,16,17,18]. It is important to note that we split the annual data into four quarterlies for practical reasons. Due to the high number of geographical units with zero frequency, more partitions would have been inconvenient. Notwithstanding, studies with higher samples could benefit from conducting a monthly or even higher resolution analysis.

This study has both strengths and limitations. On the one hand and regarding the strengths, this study has provided relevant findings about the spatio-temporal distribution of suicide-related emergency calls in a South European city. To the best of our knowledge, there are no studies available in these countries in which suicide risk has been analyzed using a small-area approach. The Southern Europe cities may show different characteristics from Northern Europe, and focusing on these cities, one could provide new evidence about the suicide behavior at the community level. In addition, a complex modeling has been used in order to improve the model fit. Previous studies have shown that the autoregressive model used here provided better results than other spatio-temporal models [29]. This model, however, is still infrequent, and this study has also provided new evidence about its possible benefits when applied to a public and social health problem compared to other spatial and spatio-temporal modeling approaches. Other alternative models could also be appropriate—for example, negative binomial models. The zero-inflated models can also be used to treat with an excess of zeros, but not in this situation, as it can not be assumed that there are some census block groups where the population are not exposed to the suicide behavior.

On the other hand, this study also has some limitations. First, results show a general landscape about the spatio-temporal distribution of suicide-related emergency calls; nevertheless, a further analysis is needed to understand the underlying processes and the covariates that could explain these patterns. Previous studies have shown that age of suicide victim, as well as gender, could be important individual characteristics. Prevalence of suicide behavior among men has also been found to be higher than the prevalence among women. Moreover, the age cohort could also affect the risk of suicide [7]. These data, however, were not available for the study. Neighborhood-level characteristics could also be included in order to understand the ecological suicide risks. Some studies have supported the relationship between neighborhood variables and different social outcomes such as family violence or crime [43,44,45,46,47,48]. Likewise, previous research has suggested that areas with lower levels of socioeconomic status, higher rates of rurality, and highly fragmented areas would show higher risks of suicide behavior in their population [9,13,25,35,49,50,51]. Future studies would benefit from analyzing these neighborhood-level covariates and their influence in the spatial variations of suicide risks. In addition, despite the benefits of autoregressive modeling, it is important to take into account the computational complexity of this kind of models, which causes a high computation time [29]. We are now exploring new possibilities and developing tools to decrease the computation time without losing complexity.

## 5. Conclusions

This study has shown the presence of small-area variations in suicide-related emergency calls and the need of including temporal terms in the analysis. A 7-year study divided into 28 trimesters has provided the insights of clear differences in the spatio-temporal effects, and also that there is a seasonal pattern. It should be noticed that the growing amount of yearly (quarterly, weekly, daily and even hourly) data available is moving researchers to use spatio-temporal models that could bring more insights about the temporal behavior and not only about the aggregated (in terms of time) spatial data. Models that incorporate these temporal components (like the one used here) are needed and should be used by researchers.

To conclude, our results may contribute to implementing strategies to prevent suicide in the community, as well as possibly being a useful tool in the suicide-related police interventions. Despite the social and economic costs of suicide in our societies, and the clear need of developing preventive actions, there is still a lack of prevention strategies and plans that could adequately face this social problem. The hotspot areas found in this study could guide police action to effectively manage its resources and develop preventive strategies to these neighborhoods with higher risks of suicide. Moreover, analyzing those neighborhoods where the risk has increased or decreased in the last years and exploring the covariates that could be explaining these changes over time could provide helpful information about suicide behavior risks in order to assess the impact of preventive policies.

## Figures and Tables

**Table 1:** Summary statistics for counts of suicide-related emergency calls by Valencia census block groups, for globally and annually aggregated data, 2010–2016.

Statistic	Global (2010–2016)	2010	2011	2012	2013	2014	2015	2016
**Counts of Suicide-Related Emergency Calls**								
Total	6537	709	824	781	968	1082	1126	1047
Min.	0	0	0	0	0	0	0	0
Max.	70	11	18	12	14	15	19	15
Mean	11.84	1.24	1.46	1.37	1.72	1.91	2.02	1.85
**Standardized Suicide-Related Emergency Calls Ratio**								
Min.	0.00	0.00	0.00	0.00	0.00	0.00	0.00	0.00
Max.	7.38	10.46	17.72	10.95	10.90	10.50	8.85	10.62

**Table 2:** Mean and standard deviation of the posterior distribution along with the 95% credible interval of the parameters of the pure spatial model.

Parameter	Mean	SD	Quantile 0.025	Quantile 0.975
μ	−0.126	0.032	−0.191	−0.070
$\sigma_{\phi }$	0.355	0.093	0.181	0.534
$\sigma_{\theta }$	0.478	0.028	0.424	0.532

**Table 3:** Mean and standard deviation of the posterior distribution along with the 95% credible interval of the parameters of the spatio-temporal model.

Parameter	Mean	SD	Quantile 0.025	Quantile 0.975
μ	−0.269	0.032	−0.334	−0.208
$\sigma_{\phi }$	0.272	0.058	0.159	0.384
$\sigma_{\theta }$	0.512	0.025	0.462	0.564
$\sigma_{\alpha }$	0.034	0.029	0.002	0.105
ρ	0.692	0.024	0.644	0.739

**Table 4:** Mean and standard deviation of the posterior distribution along with the 95% credible interval of the parameters of the spatio-temporal quarterly model.

Parameter	Mean	SD	Quantile 0.025	Quantile 0.975
μ	−0.362	0.045	−0.450	−0.275
$\beta_{1}$	−0.122	0.057	−0.230	−0.008
$\beta_{2}$	0.093	0.059	−0.024	0.208
$\beta_{3}$	0.118	0.053	0.016	0.227
$\sigma_{\phi }$	0.160	0.030	0.102	0.220
$\sigma_{\theta }$	0.359	0.019	0.323	0.398
$\sigma_{\alpha }$	0.106	0.032	0.051	0.178
ρ	0.903	0.009	0.885	0.919

## Supplementary Materials

The following are available online at www.mdpi.com/1660-4601/14/7/735/s1. Section S1: WinBUGS code for the final model. Section S2: Convergence of the parameters for the final model.
