# Supplementary material for: Spatio-Temporal Analysis of Suicide-Related Emergency Calls
Source: Int J Environ Res Public Health. 2017 Jul 6;14(7):735. doi: 10.3390/ijerph14070735 (PMC5551173; doi:10.3390/ijerph14070735)
Supplement: Supplementary file 1 [file ijerph-14-00735-s001.zip › Supplementary Material 1.pdf]

```
model{
   for(i in 1:n){
      for(t in 1:T){
         O[t,i]~dpois(eta[t,i])
         log(eta[t,i]) <- log(E[t,i]) + beta[1]*X1[t,i] + beta[2]*X2[t,i] + beta[3]*X3[t,i] +
                  mu + alpha[t] + theta.ST[t,i]
         R[i,t] <- 100*exp(beta[1]*X1[t,i] + beta[2]*X2[t,i] + beta[3]*X3[t,i] + mu +
                  alpha[t] + theta.ST[t,i])
   }

#Spatio-temporal effect for the first period

   theta.S[1,1:n]~car.normal(adj[],weights[],num[],prec.phi)
   for(i in 1:n){
         BYM[1,i]~dnorm(theta.S[1,i],prec.theta)
   }
   for(i in 1:n){theta.ST[1,i] <- pow(1-rho*rho,-0.5)*BYM[1,i]}

#Spatio-temporal effect for the subsequent periods

   for(t in 2:T){
      for(i in 1:n){
         theta.ST[t,i] <- rho*theta.ST[t-1,i] + BYM[t,i]
         BYM[t,i]~dnorm(theta.S[t,i],prec.theta)
      }
      theta.S[t,1:n]~car.normal(adj[],weights[],num[],prec.phi)
   }

#Prior distributions

   mu~dnorm(0,0.01)
   alpha[1:T]~car.normal(adj_temp[],weights_temp[],num_temp[],prec.alpha)
   prec.phi <- pow(sigma.phi,-2)
   sigma.phi ~ dunif(0,1)
   prec.theta <- pow(sigma.theta,-2)
   sigma.theta ~ dunif(0,1)
   prec.alpha <- pow(sigma.alpha,-2)
   sigma.alpha ~ dunif(0,1)
   rho~dunif(-1,1)

   for(i in 1:3){
         beta[i] ~ dnorm(0, 0.00001)
   }
}
```
