# Supplementary figures and images for: Spatio-Temporal Analysis of Suicide-Related Emergency Calls
Source: Int J Environ Res Public Health. 2017 Jul 6;14(7):735. doi: 10.3390/ijerph14070735 (PMC5551173; doi:10.3390/ijerph14070735)

$\beta_1$

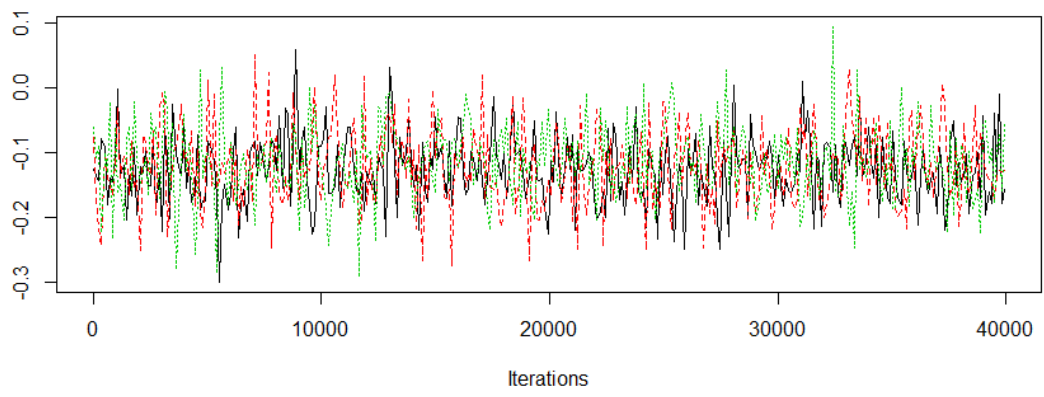

$\beta_2$

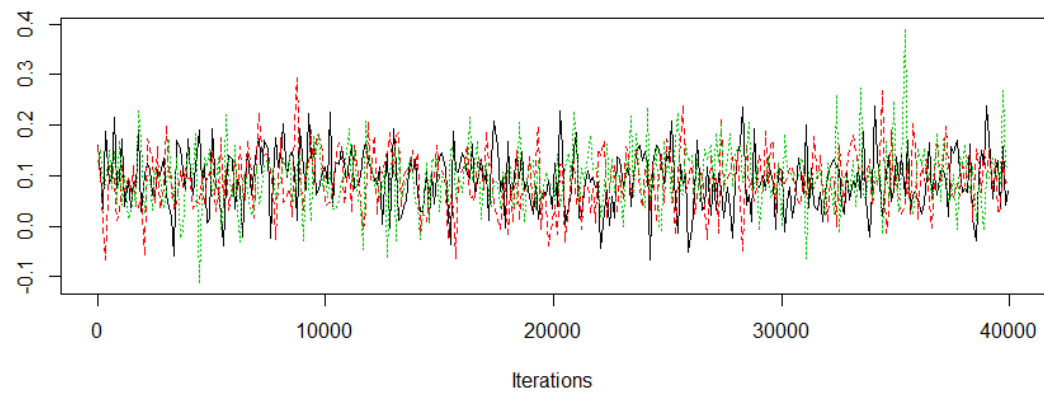

$\beta_3$

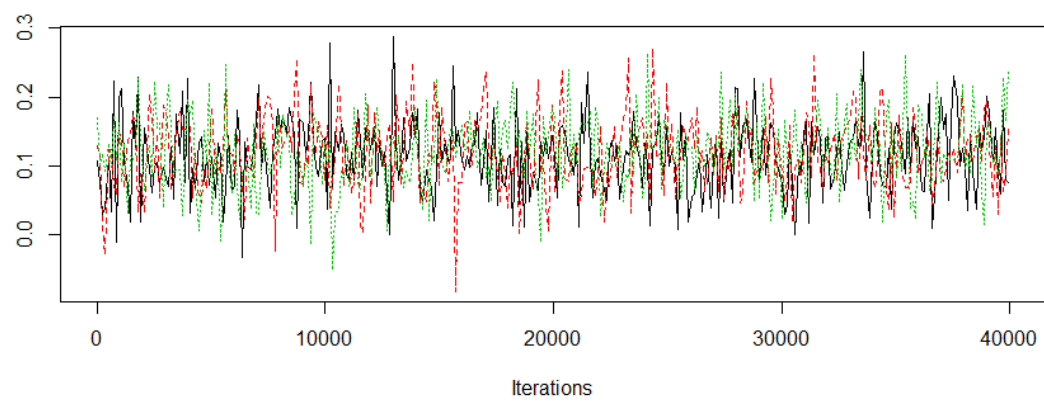

$\sigma_\varphi$

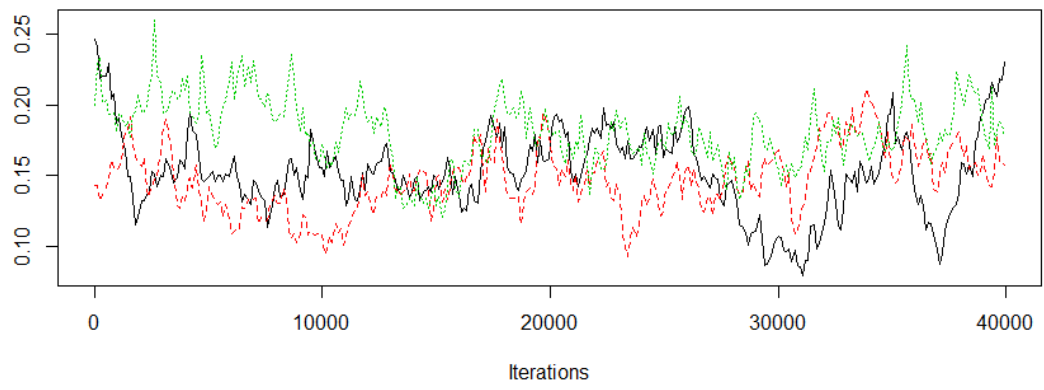

$\sigma_\theta$

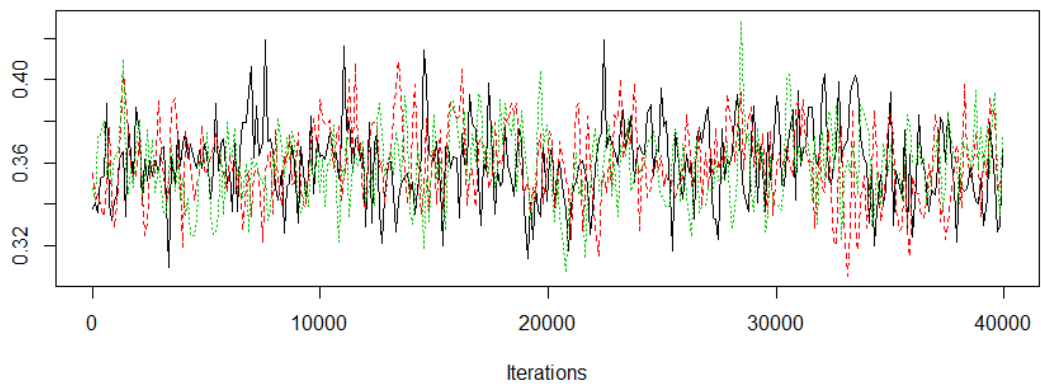

$\sigma_\alpha$

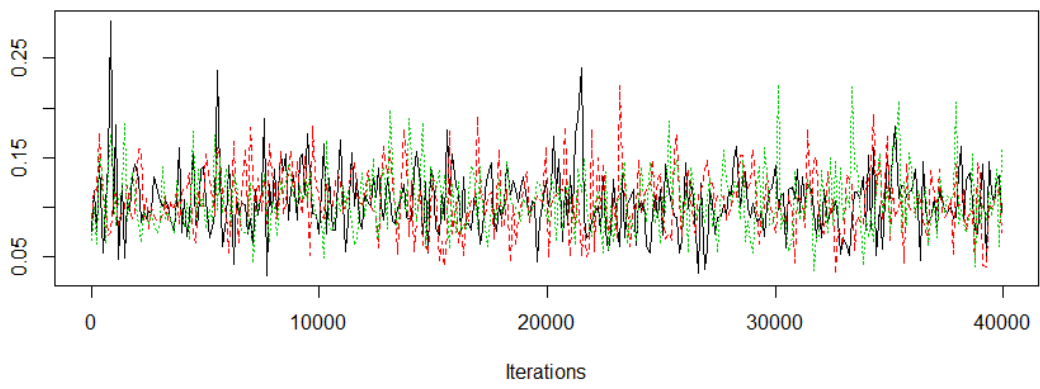

$\rho$

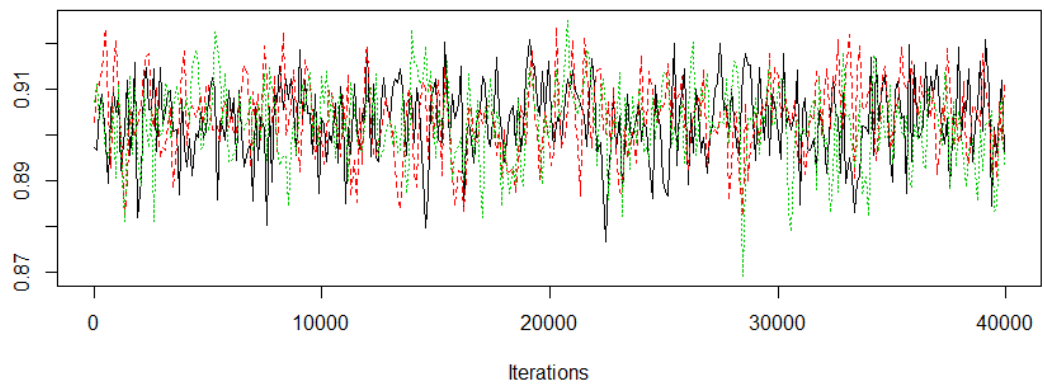

Supplement: Supplementary file 1 [file ijerph-14-00735-s001.zip › Supplementary Material 2.pdf]
